# Novel homozygous mutation in a colombian patient with persistent müllerian duct syndrome: expanded phenotype

**DOI:** 10.1590/S1677-5538.IBJU.2018.0808

**Published:** 2019-01-29

**Authors:** Mary García Acero, Olga Moreno, Andrés Gutiérrez, Catalina Sánchez, Juan Guillermo Cataño, Fernando Suárez-Obando, Adriana Rojas

**Affiliations:** 1 Human Genetic Institute Pontificia Universidad Javeriana Bogotá Colombia Human Genetic Institute, Pontificia Universidad Javeriana, Bogotá, Colombia;; 2 Department of Urology Hospital Universitario San Ignacio Bogotá Colombia Department of Urology, Hospital Universitario San Ignacio, Bogotá, Colombia;; 3 Hospital Universitario San Ignacio Bogotá Colombia Genetic Service, Hospital Universitario San Ignacio, Bogotá, Colombia

**Keywords:** Mullerian Ducts, Anti-Mullerian Hormone, Persistent Mullerian duct syndrome [Supplementary Concept], Disorders of Sex Development

## Abstract

The anti-Müllerian hormone triggers the regression of uterus and fallopian tubes in male embryos; if there are problems in the synthesis or action of this protein, Müllerian structures persist in an otherwise phenotypic male. The most frequent clinical presentation of Persistent Mullerian Duct syndrome is cryptorchidism and inguinal hernia. The few cases reported in adults are incidental findings or inguinal hernias. However, we present an adult male with history of bilateral cryptorchidism with unsuccessful orchidopexy, who presents with a large abdominal mass with the finding of a seminomatous tumor and persistence of Müllerian structures, in whom the variant c.916delC (p.Leu306Cysfs*29) in the AMHR2 gene not previously reported was documented.

## INTRODUCTION

**Figure 1:**
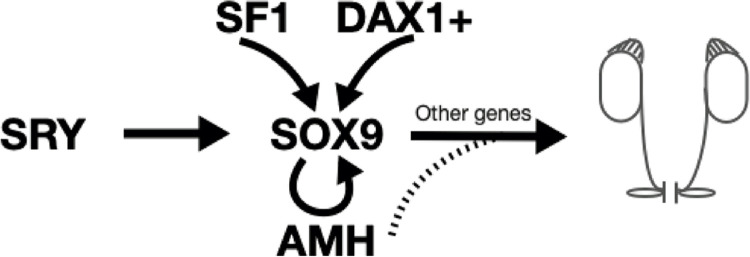
In normal XY males, the SRY gene begins testicular differentiation by interacting with other genes that determine sex, after which the differentiation and proliferation of Sertoli cells that secrete anti-Müllerian hormone (AMH) promote the regression of the Müllerian ducts and allow the differentiation of the Wolff ducts that will originate the vas deferens, the epididymis, seminal vesicles and ejaculatory ducts. SRY (Sex Determining Region Y), SOX9 (SRY-Box 9), DAX-1 (also know as NR0B1-Nuclear Receptor Subfamily 0 Group B Member 1), AMH (Anti-Mullerian Hormone).

Male sex determination is a process determined by: 1) testis formation of the primitive gonad (sex determination) through various transcription factors, and 2) differentiation of internal and external genitalia by action of hormones secreted by the fetal testicle (sex differentiation). It starts by activation of SRY gene in the precursor cells and translocate to the nucleus and binds to the enhancer region of SOX9, to intervene in the differentiation and proliferation of Sertoli cells and the tubular organization of the testis that secret anti-Müllerian hormone (AMH) to promote the regression of Müllerian ducts (fallopian tubes, uterus and upper vagina) (Figure-1) (1). Testicular descent eventually occurs until the end of pregnancy and is both dependent and independent of hormones.

AMH is a glycoprotein secreted by the Sertoli cells of the testes from the moment of sexual differentiation until puberty. The AMH gene, a member of the transforming growth factor-𝛽 (TGF-𝛽) is activated by SRY but also by genes upstream and downstream as SOX9, SF-1, WT-1 and GATA415 (2-5). The defects of synthesis and action of AMH also known as Persistent Müllerian Duct Syndrome (PMDS), is an unusual disorder of sex development with autosomal recessive inheritance. In 52% of cases it happens because of mutations in the gene that encodes for anti-Müllerian hormone (AMH) and less frequently for mutations in the AMH receptor, type II (AMHR2) causing hormonal resistance (6). Mutations within these two genes have been detected in 85% of patients with PMDS, with unknown genes in the remaining 15% of cases.

AMHR2 is a membrane protein, which contains a N-terminal extracellular domain that binds AMH, a transmembrane domain, and an intracellular domain with serine/threonine kinase activity. The AMHR2 gene is located at 12q13 and contains 11 exons. The extra cellular domain of AMH binding is encoded by the first 3 exons (5); the exon 4 encodes the transmembrane domain, and the catalytic intracellular serine/threonine domain is encoded by the last 7 exons (7).

The incidence of PMDS is low, with near 200 cases documented in the world. Individuals with PMDS are phenotypical male with karyotype 46, XY with presence of Wolffian and Müllerian structures and normal virilization, consequently with normal external male genitalia. However, some individuals have infertility secondary to prolonged cryptorchidism or anomalies in the communication between the testis and the male ducts (epididymis and upper deferent duct) secondary to presence of Müllerian ducts attachments to testes preventing the normal descent. According to previous case reports, delayed diagnosis at adulthood may cause infertility (5).

The determination of serologic levels of AMH is useful when it is suspected in childhood (undetectable with mutations in AMH or normal-elevated with mutations in AMHR2) (8, 9), with certain limitations in adults because AMH levels are low and cannot discriminate between AMH and AMHR2 mutations, neither in cases of PMDS not associated with mutations in these genes. The most common symptoms of PMDS are inguinal hernia, undescended testis, abdominal mass and less frequently testes tumor (10). Also, Müllerian structures could be discovered as an incidental finding during an abdominal surgery or imaging. The treatment in men with PMDS is still under debate. Previous papers have suggested that surgery should be conducted in separate procedures: first, testis reposition into the scrotum with hernia repair and testis biopsy and second, orchidectomy upon indication for atrophic testis or when orchidopexy cannot be performed (10, 11). As in non-descended gonads, the testes of patients with this syndrome have a high risk of developing malignant tumors, calculated between 15 and 18% (12). Tumors of all types have been reported: embryonal carcinoma, teratomas, embryonal sac tumors (Yolk Sac), choriocarcinomas and seminomas. A total of 30 cases of malignant transformation have been identified in patients with PMDS (13).

Recently, we detected a mutation not previously reported in the AMHR2 gene in a male with congenital cryptorchidism with failed orchidopexy and unsuccessful surveillance who arrived with an abdominal mass corresponding to a seminomatous tumor.

## MATERIALS AND METHODS

### Patient

A Colombian 33-year-old married man, son of consanguineous parents, with history of orchidopexy at 4 years, without follow-up after surgery to evaluate the testicular position, was recently admitted to our clinic with complaints of urinary symptoms. Physical examination showed that the patient was phenotypically male with bilateral cryptorchidism and normal penile length. His secondary sex characteristics were normal but an abdominal mass was palpated in the hypogastrium; therefore, additional tests were done. The patient had a personal history of infertility not studied.

The hormonal evaluation of the patient at the time of consultation revealed hypergonadotropic hypogonadism FSH (2.2mUI/mL-reference: 0.2-1.4), LH (0.08ng/mL-reference: 0.01-0.5), testosterone (0.04ng/mL-reference: 0.01-0.04-: basal without alterations) and AMH: 816pg/L (reference: 360-668pg/L). Other laboratory results showed elevated tumor markers alpha-fetoprotein and human chorionic gonadotropin. The pelvic MRI showed a heterogeneous mass of tumoral aspect in the pelvis, extension to the hypogastrium and retrovesical space, with a volume of 640cc. Features of the mass suggested a mixed lesion with solid and multilocular cystic component, with some hemorrhagic foci, which contacts the dome of the bladder and base of the prostate, displacing the seminal vesicles to the left, with suspicious image of Ewing’s sarcoma.

**Figure 2:**
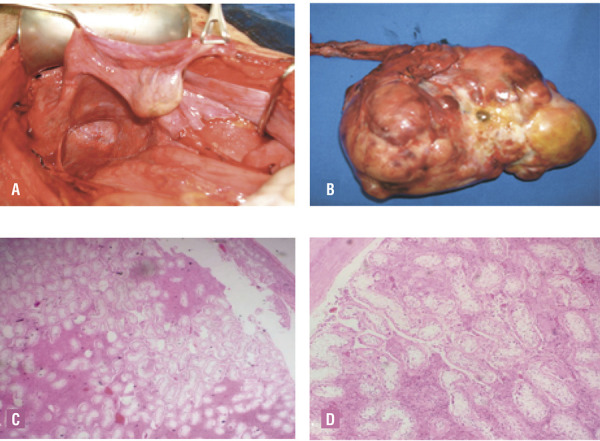
Intraoperative appearance and histopathologic findings.

Diagnostic laparotomy and resection of the mass revealed a 20cm multi-loculated mass arising from the left undescended gonad. Right hypotrophic undescended gonad was adhered to Müllerian ducts (Figure-2). Total excision of the mass, uterus and bilateral fallopian tubes gonads was performed. Pathologic examination revealed pure seminomatous tumor and right atrophic testis (Figure-2).

**A)** Left intra-abdominal gonad associated with tubular structure reminiscent of uterine horn and broad ligament, **B)** multiloculated mass dependent of left gonadal tissue, **C)** pure seminomatous tumor, and **D)** atrophic tissue of right teste.

Sections from both fallopian tubes showed congestion and fibrosis. No ovarian tissue was seen. The patient received multidisciplinary management by endocrinology, urology, genetics, oncology and psychology. His family received genetic counseling for the risk of recurrence and the carrier status.

## METHODS

### Cytogenetic

Cytogenetic analyses of the patient were carried out based on phytohemaglutinin-stimulated peripheral blood lymphocyte cultures, according to standard laboratory protocols. Chromosome preparations were treated with HCl and stained with Wright for banding G. A total of 50 metaphase cells were analyzed at the 550-band resolution level.

Molecular cytogenetics using fluorescence in situ hybridization (FISH) with the following probes and probe sets was subsequently performed following the manufacturer’s instructions: SRY (in Yp11.31; Cytocell-Aquarius, Cambridge, UK), DYZ1 (in Yq12; Cytocell-Aquarius, Cambridge, UK). Also, a control probe for Xp11.1-q11.1 was hybridized. As counterstain 4’,6-diamidino-2-phenylindole DAPI was used. 200 metaphases and 50 nuclei were analyzed with the ZEISS ZEN© microscope software.

### Molecular analysis of the AMH and AMHR2 gene

Genomic DNA was extracted from the peripheral blood of the proband, following standard salting-out protocol. DNA was amplified by PCR using specific primers for the AMH and AMHR2 genes, reported by Yumie et al. (14) (Table-1). PCR conditions were: one cycle of 95ºC for 5 minutes, 35 cycles of 97ºC for 45 seconds; annealing temperatures for 45 seconds; 72ºC for 2 minutes; and a final cycle of 72ºC for 10 minutes. The PCR products were analyzed by direct DNA sequencing on an ABI 310 sequencer (Applied Biosystems; Thermo Fisher Scientific, Inc., Waltham, MA, USA). The results were analyzed by comparing the sequences with those reported in Gene Bank NM_000479 and NM_020547, respectively.

**Table 1:** Primer sequences and annealing temperatures of fragments of AMH and AMHR2.

Gene exon	Forward primer (5’ – 3’)	Reverse primer (5’ – 3’)	Annealing Temp (Cº)
AMH 1	F – AAACACCCCACCTTCCACTC	R –CCGGCCCACCTGAAGGAA	60
AMH 2	F – CAGGGACAGATCCCAAAGAT	R – TACTGCAGACCCTGCAACAA	60
AMH 3 – 4	F – GTAGAGCGGGGCTGGGTA	R – CGCAATTGGAGGAGTTGAGA	57
AMH 5	F – CTGGACACCGTGCCCTTC	R – TGGGGTCCGAATAAATATGG	57
AMHR2 1 – 2	F – CAGGATGCCCTGTATCTGAAG	R – acaccccaggatgtgtctgt	58
AMHR2 3 – 4	F – CTCTGTTTCCACACCCCATT	R – GGAGAGGGGTCAGAGCTTTT	58
AMHR2 5 – 6	F – GACTCCCATGACCTCTCACAA	R – CATGTAGCCCCCACCTCTAT	58
AMHR2 7	F – GGATGGATCAGCCGTCTC	R - AGGCAGAATCACAAACATAGCA	61
AMHR2 8 – 9	F - AAAAAGAGGGAGGAAGAAAATC	R – ttggggtgaacctagaatgg	54
AMHR2 10	F – CCCTTTCTACATGGTAGGCA	R – ACGTCCTTGAAGCCCATGCCCA	49
AMHR2 11	F – TTTTAACCCTGGGGCCCACT	R - GCACACCTACCCCAAGTCAC	58

## RESULTS

**Figure 3:**
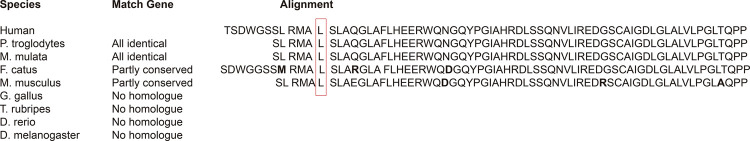
Conservation of AMHR2 protein between different species. The red box indicates the altered amino acid in the individual reported conserved in all species in which this protein is present. Bold letters indicate amino acid changes with respect to the human Wild type sequence.

Patient’s karyotype showed 46.XY.ish Yp11.31 (SRY+). Sanger sequencing revealed the existence of a homozygous variant NM_020547 (AMHR2) c.916delC (p.Leu306Cysfs*29) in the 7 exon of the AMHR2 gene, that produces a change in the amino acid that results in a pathogenic frameshift with a shorter protein of 334 amino acids, respect to the normal protein of 574 amino acids, removing a significant part of the intracellular serine/threonine kinase domain responsible of biological activity. This variant has not been previously reported. The amino acid Leucine is conserved in some mammal species (Figure-3), indicating that changes in this position can have a significant effect on the protein.

Although this variant has not been reported previously in the dbSNP, 1000G, and ExAC databases, it has been identified as pathogenic by bioinformatics analysis tools Protein Variation Effect Analyzer (provean.jcvi.org/index.php) and Polyphen (http://genetics.bwh.harvard.edu/pph2/).

## DISCUSSION

To date, 4 patients with PMDS have been reported in Colombia: two children with diagnosis during laparoscopic treatment of cryptorchidism without molecular study, (15) and two adult patients with cryptorchidism and inguinal hernia were identified in pelvis MRI with uterus and fallopian tubes (16).

This case report highlights the importance of a continuous and multidisciplinary evaluation of patients with cryptorchidism, a clinical spectrum of disorder of sexual differentiation, whereas the post-operative follow-up of the patient would have contributed to the timely management of the failed orchidopexy and to avoid secondary consequences as tumor and infertility.

The initial laboratory evaluation in a phenotypically male with bilateral nonpalpable testes must include karyotype, ultrasonography of the pelvic structures, serum testosterone, gonadotropins (LH, FSH), anti-Müllerian hormone level, and exploratory surgery is usually necessary for definitive diagnosis. In males, serum levels of AMH remain high until 2 years of age and persist in measurable levels until puberty, before decreasing to undetectable levels at puberty (17).

AMH is a possible screening assay in young patients with bilateral cryptorchidism, due to the possibility of differential diagnosis of PMDS, even more in the presence of a positive history of consanguinity, bilateral cryptorchidism and male infertility, as in this case, which was susceptible to be diagnosed in childhood. An opportune diagnosis allows an adequate surgical management, and giving advice in the surveillance of the tumoral risk and in the possible compromise of the fertility, but even the molecular diagnosis allows confirming the diagnosis and providing genetic family counseling.

Mutations in the intracellular domain, like this, have been reported previously, all with lack of the kinase activity (7, 18) and alteration of the secondary catalytic activity, therefore, without functional AMH that leads to regression of the Mullerian ducts.

Likewise, the importance of long-term postoperative follow-up of orchidopexy is highlighted, due to possible complications associated with non-descendant testicular tissue (eg, testicular cancer, occult testicular torsion, infertility) as occurred in this patient, who, due to lack of knowledge and non-adherence to periodic follow-ups, had a late diagnosis of PMDS and testicular tumor.

## CONCLUSIONS

In conclusion, PMDS is a condition that is observed in men with cryptorchidism and/or inguinal hernia. It must be diagnosed and treated promptly to protect the fertility and prevent the potential risk of tumors. Our results expand the mutational spectrum of this infrequent condition and emphasize that PMDS should be included in the differential diagnosis of cryptorchidism. Genetic counselling should be considered in cases of parental consanguinity.
